# Gas explosion early warning method in coal mines by intelligent mining system and multivariate data analysis

**DOI:** 10.1371/journal.pone.0293814

**Published:** 2023-11-02

**Authors:** Hongxia Li, Yiru Zhang, Wanli Yang

**Affiliations:** 1 College of Management, Xi’an University of Science and Technology, Xi’an, Shaanxi, China; 2 College of Energy Engineering, Xi’an University of Science and Technology, Xi’an, Shaanxi, China; 3 Xi’an University of Science and Technology, Xi’an City, Shaanxi Province, China; 4 Shaanxi Xixian Financial Holdings Group Co., Ltd., Xianyang, Shaanxi, China; University 20 Aout 1955 skikda, Algeria, ALGERIA

## Abstract

In order to predict gas explosion disasters rapidly and accurately, this study utilizes real-time data collected from the intelligent mining system, including mine safety monitoring, personnel positioning, and video surveillance. Firstly, the coal mine disaster system is decomposed into sub-systems of disaster-causing factors, disaster-prone environments, and vulnerable bodies, establishing an early warning index system for gas explosion disasters. Then, a training set is randomly selected from known coal mine samples, and the training sample set is processed and analyzed using Matlab software. Subsequently, a training model based on the random forest classification algorithm is constructed, and the model is optimized using two parameters, Mtry and Ntree. Finally, the constructed random forest-based gas explosion early warning model is compared with a classification model based on the support vector machine (SVM) algorithm. Specific coal mine case studies are conducted to verify the applicability of the optimized random forest algorithm. The experimental results demonstrate that: The optimized random forest model has achieved 100% accuracy in predicting gas explosion disaster of coal mines, while the accuracy of SVM model is only 75%. The optimized model also shows lower model error and relative error, which proves its high performance in early warning of coal mine gas explosion. This study innovatively combines intelligent mining system with multidimensional data analysis, which provides a new method for coal mine safety management.

## Introduction

With the continuous advancement of industrialization, coal mines play a vital role as an important energy supply source. However, due to the complex geological conditions and high-risk working environment within coal mines, mine accidents occur frequently, especially coal mine explosion accidents, posing significant threats to human lives and property safety [1]. In order to enhance the early warning capability of coal mine explosion accidents, many researchers have been devoted to developing new methods and technologies. Intelligent mining systems and multivariate data analysis, as emerging research areas in recent years, have great potential to improve the effectiveness of coal mine explosion early warning [2].

An intelligent mining system is a system that utilizes computer technology and artificial intelligence algorithms to automatically analyze and process large-scale data. It can extract valuable information from massive coal mine data and establish reliable warning models through techniques such as data mining, pattern recognition, and machine learning [3]. Compared to traditional warning methods, intelligent mining systems can more accurately predict the occurrence probability and risk level of coal mine explosion accidents [4]. Multivariate data analysis refers to the analysis and integration of various types of data sources to obtain more comprehensive and accurate information [5]. In the coal mine explosion warning, not only the data related to the mine itself, such as gas concentration and temperature, need to be considered, but also other relevant factors, such as meteorological conditions and personnel density [6]. By analyzing and integrating these multivariate data, potential hazard factors can be identified, thereby improving the accuracy and timeliness of early warning.

This study aims to investigate the coal mine gas explosion early warning method based on intelligent mining systems and multivariate data analysis. Firstly, the coal mine disaster system is subdivided into sub-systems of disaster-causing factors, disaster-prone environments, and vulnerable bodies to establish an indicator system for gas explosion disaster early warning. Secondly, a training set is randomly selected from known coal mine samples, and these training samples are processed and analyzed using Matlab software to construct a training model based on the random forest classification algorithm. The performance of the model is optimized by adjusting two parameters, Mtry and Ntree. Finally, the constructed random forest-based coal mine gas explosion early warning model is compared with a classification model based on the support vector machine (SVM) algorithm, and specific case analyses are conducted to validate the effectiveness of the optimized random forest algorithm.

## Literature review

Coal mine explosions are among the most serious accidents in coal mine safety, posing significant threats to the lives and property of miners. Therefore, the discussion on coal mine explosion early warning methods is of great significance [7]. In recent years, with the continuous development of intelligent mining systems and multi-dimensional data analysis techniques, significant improvements and advancements have been made in coal mine explosion early warning methods.

In previous studies, many researchers have utilized intelligent mining systems and multi-dimensional data analysis techniques to study coal mine explosion early warning. For instance, Demirkan et al. (2022) proposed a coal mine explosion early warning method based on machine learning algorithms. By collecting and analyzing on-site data, they established a predictive model that can monitor the safety status of coal mines in real-time and issue timely warning signals [8]. Dursun (2020) introduced a coal mine explosion early warning method based on neural networks. By monitoring and analyzing environmental data in coal mines, he used a neural network model for modeling and prediction, achieving effective early warning for coal mine explosions [9]. Demirkan et al. (2022) analyzed the data from various sub-systems of a gas accident monitoring and early warning system. When the excessive gas concentration was detected, timely alarms were issued to underground workers, enabling them to take appropriate measures to mitigate the risk of gas explosions. Through analysis and research on the system’s functionality, they proposed an overall construction approach and optimized the individual sub-systems to ensure the system’s comprehensive effectiveness [10]. Chen et al. (2022) employed a Bayesian network as a research method and integrated expert knowledge and data learning to construct a Bayesian network risk recognition model for coal mine gas explosions. They identified and assessed the risks of gas explosion accidents, identified potential risk sources leading to accidents, and performed a warning analysis of disaster accident levels based on setting accident risk warning intervals using Bayesian networks [11]. Cai et al. (2021) thought that applying machine learning technology to the prediction and early warning of coal mine gas concentration could effectively prevent the occurrence of gas explosion accidents [2]. Li et al. (2023) used Radial Basis Function (RBF) neural network to build a prediction model of roof instability under repeated mining. Compared with the general RBF neural network prediction model, the correlation coefficient between the predicted value of the research model and the actual value of the output index was greater than 0.05 [12]. Thilagavathi et al. (2023) used the comprehensive activities of temperature, tension and gas sensors and the IoT module to identify the temperature, strain and climate in the coal mine shaft, and recorded all the information into the cloud by using the information log to establish a coal mine safety monitoring system [13]. Kong et al. (2023) proposed an anomaly detection method based on dynamic threshold and depth self-coding Gaussian mixture model [14].

However, despite some progress, current research still has some shortcomings. Existing studies mainly focus on the analysis of single data sources, neglecting the comprehensive utilization of multi-dimensional data. Moreover, existing early warning methods have certain rates of false and missed alarms in practical applications, necessitating further improvement in the accuracy and reliability of early warnings. The novelty of this study lies in the research on coal mine explosion early warning methods based on intelligent mining systems and multi-dimensional data analysis. Multiple data sources will be utilized, including coal mine environmental data, sensor data, and personnel behavior data. These data will be collected, cleaned, and integrated using intelligent mining systems. A comprehensive predictive model will be established by employing multi-dimensional data analysis techniques to achieve accurate early warning of coal mine explosions.

## Construction of coal mine early warning model based on intelligent mining systems and multi-dimensional data analysis

### Development of coal mine disaster system evaluation indicator system

In recent years, coal mine gas explosions have been the result of the explosive oxidation reaction that occurs when the released gas in the mine gallery combines with the air and particulate coal powder [15, 16]. The occurrence of this disaster phenomenon requires the simultaneous satisfaction of three conditions, as shown in Table 1.

**Table 1:** Three conditions of gas explosion.

Conditions for the gas explosion	The causes of gas explosion
**Gas content**	When the gas content surpasses 9.5%, a complete reaction between the gas and oxygen in the mixed air ensues, releasing a significant amount of heat and the most potent form of explosion reaction [17].
**Adequate oxygen**	A gas explosion reaction requires a specific concentration of oxygen, defined by the volume proportion of oxygen within the mixture of gas and coal mine air, falling within a certain range [18].
**A high-temperature source of ignition**	Given the presence of an ignition source possessing ample energy, a temperature exceeding 650°C, and a combustion duration that satisfies the explosion induction period, coupled with the attainment of critical gas and air content within the air-gas mixture, there exists a high likelihood for an explosion reaction to transpire [19].

In Table 1, gas concentration, oxygen concentration, and high-temperature ignition source are necessary conditions for gas explosion reactions and important reference indicators for predicting and controlling coal mine gas explosion disasters. The research comprehensively examines the construction, attributes, and environment of the disaster system and explores the complex correlations among various causal factors in the disaster system from the perspective of sub-systems [20]. A disaster is not a simple isolated system but rather a final disaster system formed by the combination and association of multiple interacting disasters, which are initially combined into disaster sub-systems. Each disaster system has its unique construction, attributes, and environment. From a structural perspective, different types of elements and disaster accidents collectively constitute the entire disaster system, and disasters with similar attributes form their respective smaller sub-systems within the disaster system [21–23]. Simultaneously, this study provides the following explanation for the risk of gas explosion disasters: There are complex relationships and interactions among the sub-systems of causative factors, the environment conducive to disaster occurrence, and the vulnerable elements within the coal mine disaster system. Under specific conditions, these characteristic indicators collectively contribute to the probability of gas explosion accidents occurring in the mine and the resulting severity of the disaster.

The essence of coal mine gas explosion disaster risk assessment lies in determining the risk levels of potential gas explosions in coal mines. This process requires multilevel evaluation criteria, such as determining evaluation factors and specifying evaluation standards. Considering the complexity of coal mine disasters involving numerous factors, the theory of disaster systems is introduced to preliminarily select appropriate evaluation factors. Rough set attribute reduction is applied to refine the indicators, followed by the selection of rational grading criteria. This approach enables the construction of a suitable coal mine gas explosion disaster risk assessment indicator system. The principles for selecting evaluation indicators in coal mine disaster system assessment are presented in Table 2.

**Table 2:** Principles for selecting evaluation indicators.

Principle	Specific requirement
**Scientificity**	It is essential to consider the fundamental characteristics of the region where coal mine samples are obtained as the foundation for the evaluation process. This entails careful consideration of the geological structure and social attributes that may exert influence. Consequently, it is imperative to select evaluation indicators in a scientific and rigorous manner.
**Independence**	The selection of evaluation indicators should prioritize those that possess mutual independence, thereby reducing the interdependence among evaluation indicators and facilitating quantitative analysis of the significance attributed to gas explosion evaluation indicators.
**Operability**	Moreover, it is crucial to establish quantitative criteria or standards for the selected evaluation indicators.

**Fig 1:**
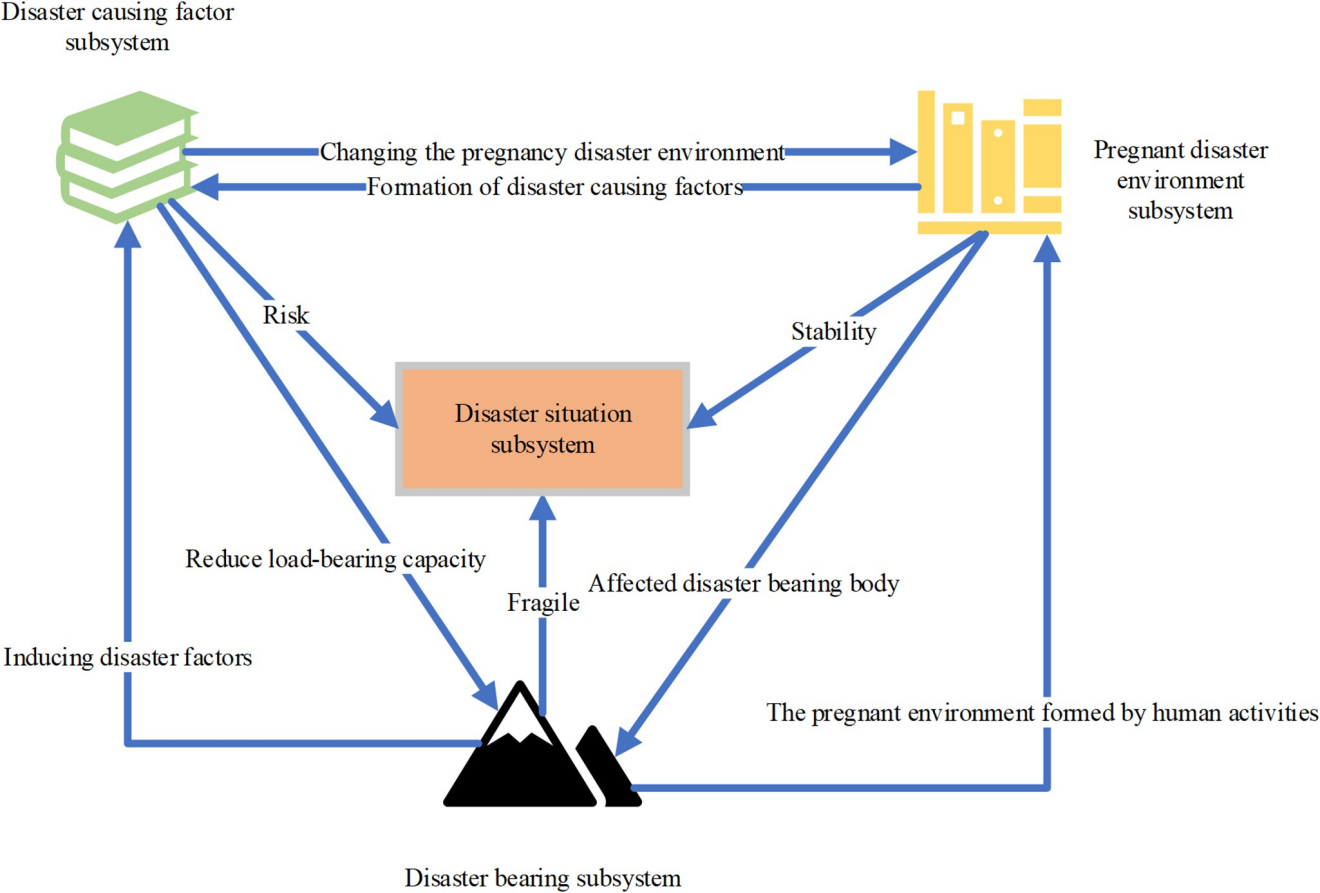
Relationships among sub-systems in the gas explosion disaster system.

Given the broad coverage and complex causal mechanisms of gas explosion disasters, it is necessary to establish a set of indicator systems based on the theory of disaster systems to accurately evaluate the essence of coal mine gas explosions from a holistic perspective. An appropriate evaluation indicator system is crucial for ensuring the accuracy of gas explosion disaster risk assessment [24]. By introducing the theory of disaster systems into the coal mine gas explosion disaster system, the conditions for gas explosion occurrence can be divided into the following three secondary indicators: the factors that lead to the triggering of gas explosions, the environment that fosters gas explosion occurrences, and the human settlements or areas covered by the affected zone, which incur social and property losses. These correspond to the concepts of causative factors, pregnant environments, and vulnerable bodies in the theory of disaster systems. The interactions among these three secondary evaluation indicators determine the severity level of gas explosion disasters. The constructed coal mine gas explosion disaster system can objectively reflect the resonant relationships, interactions, and couplings among the risk factors of gas explosion disasters, ultimately leading to corresponding levels of risk. The resonant relationships among sub-systems in the gas explosion disaster system are illustrated in Fig 1.

Based on Yan et al.’s (2022) study on coal mine safety risk, this study analyzes the characteristics and inducing factors of typical geological disasters in mines, and holds that mine geological disasters and environmental geology will have cumulative effects, and the interaction between disasters was likely to form a chain reaction, which will have a serious impact on mining areas and surrounding areas [25]. Based on the theory of disaster system, the risk assessment index system of coal mine gas explosion is constructed. Specifically, using the method of disaster system theory, the risk assessment of coal mine gas explosion disaster is decomposed into three main aspects: the danger of risk factors, the stability of potential disaster environment, and the vulnerability of fragile victims. These aspects are identified as the secondary evaluation indicators for evaluating the risk of gas explosion disaster in coal mines. Based on these secondary evaluation indicators, the corresponding three-level evaluation indicators are selected. This selection process is based on the professional knowledge in the field of coal mine safety, domestic and foreign coal mine safety regulations and standards. According to this information, the most suitable three-level evaluation index is selected [26]. The indicator system constructed in this study is shown in Table 3:

**Table 3:** Indicator system for risk assessment of coal mine gas explosion disasters.

Primary indicators	Secondary indicators	Third level indicators
**Coal mine gas assurance disaster risk**	Stability of the pregnant disaster environment	Roof stability
		Coal dust explosion hazard
		Fan operation stability
		Intactness rate of fans and air ducts
		The rationality of ventilation methods
		Qualified rate of air volume at the location where the air is used
		Reliability of monitoring device operation
		Intactness rate of gas discharge facilities
	The danger of disaster-causing factors	Gas content in the working environment
		Absolute gas emission
		Gas extraction compliance rate
		Spontaneous ignition period of coal seams
		Qualified rate of goods stacking
		Noise level
		The authenticity of the situation report
		Intensity of safety education and training
		The implementation rate of safety responsibility
		Qualified rate of technical personnel operation
		Regulatory efforts
	Vulnerability of disaster-bearing bodies	Self-rescue device allocation rate
		The installation rate of explosion-proof and explosion-proof facilities
		Timeliness of mine rescue
		Correct use of protective equipment
		Reliability of the positioning device for personnel entering the well

Table 3 decomposes the coal mine gas explosion disaster risk assessment system into three key subsystems: the stability of the pregnant disaster environment, the danger of disaster-causing factors, and the vulnerability of disaster-bearing bodies. Furthermore, the index in each subsystem is divided and quantified to determine the risk level of each index. This process constitutes the core of gas explosion disaster risk assessment index. After assigning numerical values to each index, the relative number is chosen to define the meaning and numerical range of feature attributes, and the risk level of the selected features is divided.

It is assumed that the capacity of the whole coal mine sample set is *n*, and the dataset *S* = [*s*_1_, *s*_2_, ⋯, *s*_*n*_]^*T*^ is established, in which each coal mine sample *S*_*i*_ = [*s*_*i*1_, *s*_*i*2_, ⋯, *s*_*im*_, *u*_*i*_]^*T*^ contains a final accident risk grade *u*_*i*_ and the risk level vector *U* = [*u*_1_, *u*_2_, ⋯, *u*_*n*_]^*T*^. In order to clearly define the risk level cut-off, point of each indicator, *v*_*i*_ (*k*) (*k* = 1, 2, ⋯, *n*) is introduced as the corresponding vector of the risk level cut-off point *x*_*i*_(*k*) of the *k*th indicator, and the calculation of indicator data *x*_*ij*_ is shown in Eq 1:

sij′=vik,si<sikvik+1+vik-vik+1sik+1-siksik+1+sik,sik+1,sik+1<sisik<si<sik+1 (1)

After the standardized processing of all coal mine sample data, according to the significance, scope and principle of assigning each index, considering the characteristics of mine operation and the factors affecting its safety production, combined with general grading standards and expert opinions, comprehensive consideration is made. In this study, the evaluation set of gas explosion disaster risk level in coal mine was established, *P* = {Safety; Safer; General safety; Less safe; Unsafe}, including 5 review levels. Each level corresponds to a corresponding security level vector (5, 4, 3, 2, 1). This set of methods and steps, combined with systematic analysis and professional advice, is helpful to accurately evaluate the risk of gas explosion disaster in coal mines, and turn it into an understandable safety level evaluation, which provides effective support and guidance for coal mine safety production.

### Evaluation model of gas explosion disaster risk based on random forest

The Random Forest algorithm is based on the Bagging algorithm, and the algorithm randomly selects samples with replacements to obtain the initial sample dataset used for classification in the decision trees of the Random Forest. Finally, multiple decision trees are integrated to form the Random Forest [27, 28]. It can be inferred that the decision tree is the base classifier of the Random Forest, and the Bagging algorithm embodies the idea of ensemble learning in the Random Forest. Those two algorithms are indispensable components of the Random Forest [29].

**Fig 2:**
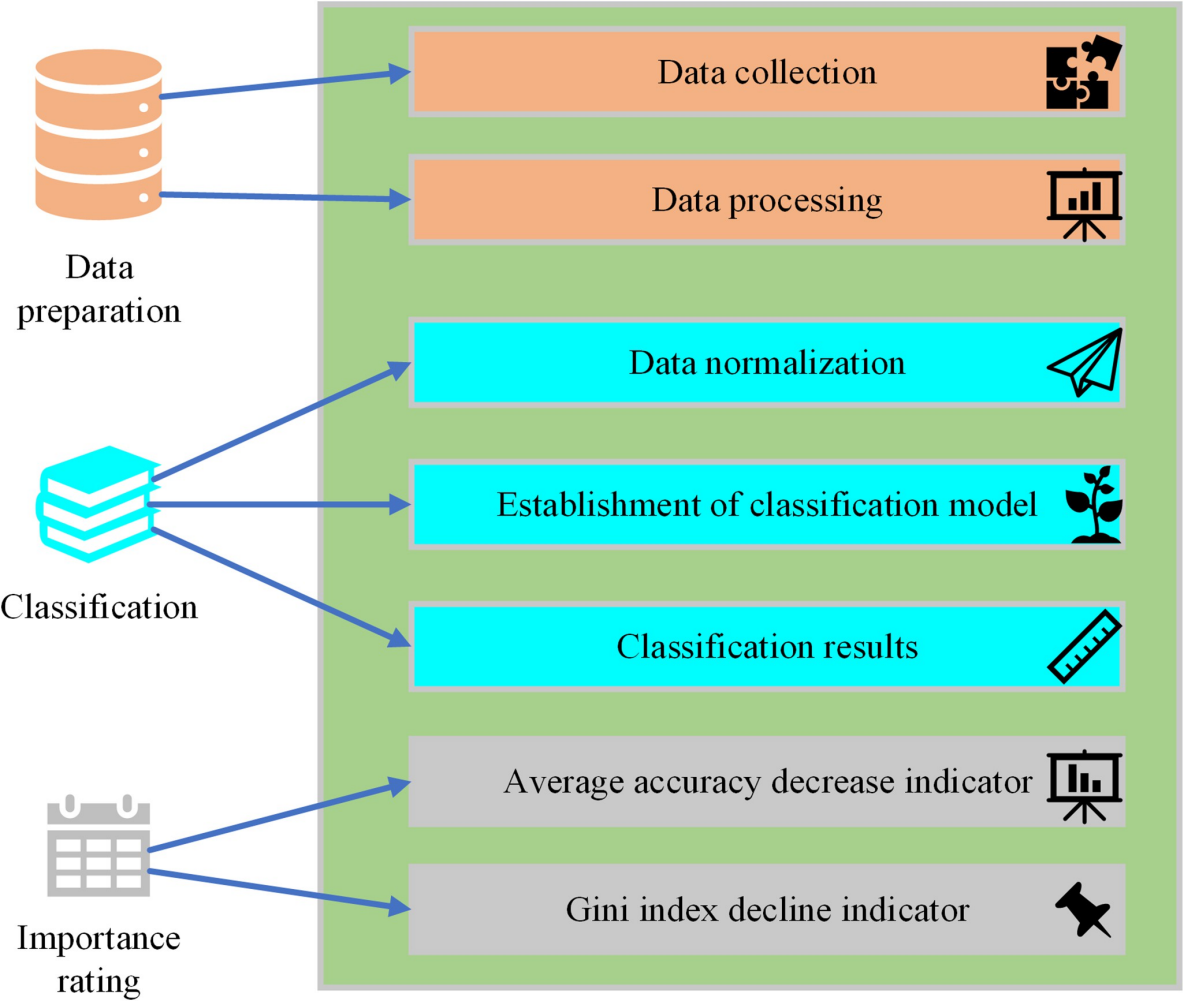
Preliminary process of constructing the random forest model.

**Fig 3:**
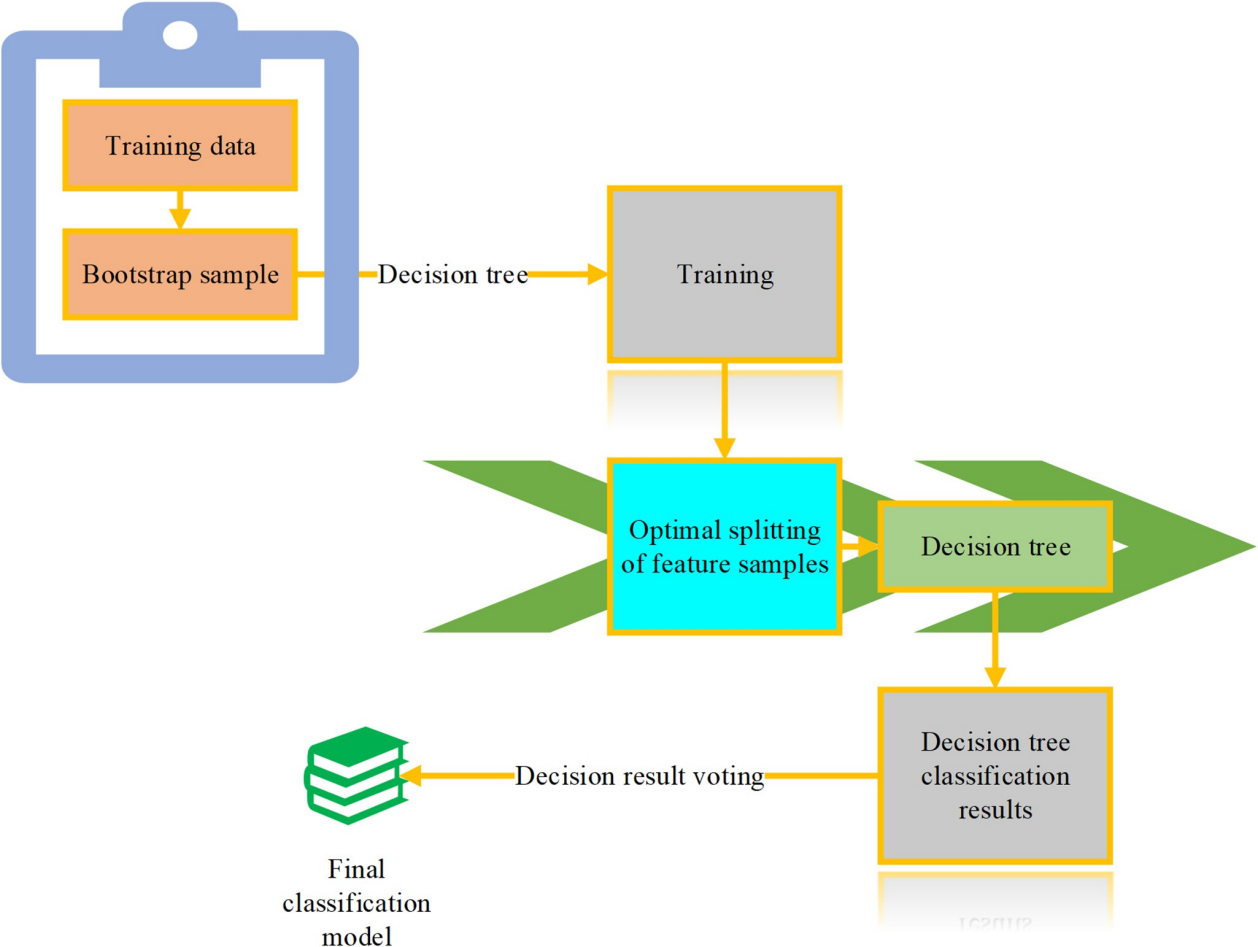
Process of establishing the random forest algorithm.

The elemental analysis of the corresponding sub-systems of the gas explosion disaster system is conducted by the theory of disaster systems. Based on the determined risk indicators for gas explosion disasters, the Random Forest is used to train the classification model and assign importance scores to the evaluation indicators. The preliminary process of constructing the specific model is illustrated in Fig 2:

Multiple decision trees can be easily combined to form a multi-classifier, which improves the limitations of a single decision tree as a classifier [30]. Therefore, multiple decision trees can be constructed to address the deficiencies of a single decision tree. This method has lower accuracy requirements for individual classifiers. After the construction process, the classification results of all the individual classifiers are tallied, and the result with the highest number of votes is selected as the outcome. The core steps in constructing the Random Forest algorithm include decision tree construction, forest establishment, voting prediction, and classification model development. The specific algorithmic process is illustrated in Fig 3:

The generation of multiple independent decision trees is a crucial first step in constructing a random forest. It is essential to ensure that each individual classifier has its independent subset of coal mine data. The Bagging algorithm is applied to process the coal mine dataset of the random forest, resulting in multiple independent subsets of coal mine samples. In the processing of the coal mine sample dataset, the concept of the Bagging algorithm is introduced, where random sampling of the dataset generates X independent small datasets. Consequently, X-independent decision trees are constructed, and these X-decision trees are integrated into a random forest without pruning. The two most important steps in constructing the random forest are the selection of the splitting attribute and the partitioning of the coal mine sample set within a node.

### Parameter optimization of the gas explosion disaster risk evaluation model

Parameter optimization is a commonly used approach to model optimization. The default parameters are used in the MATLAB implementation of the random forest algorithm. However, parameter adjustment can significantly optimize the model for the random forest algorithm [31–33]. The ability to prevent overfitting and the accuracy of the training set directly determine the classification accuracy of the random forest algorithm. Therefore, when adjusting the parameters, this study needs to consider both the ability of the random forest algorithm to prevent overfitting and its accuracy on the training set. Among them, two parameters, Mtry and Ntree, are preferred choices for optimization in the random forest algorithm [34, 35].

**Fig 4:**
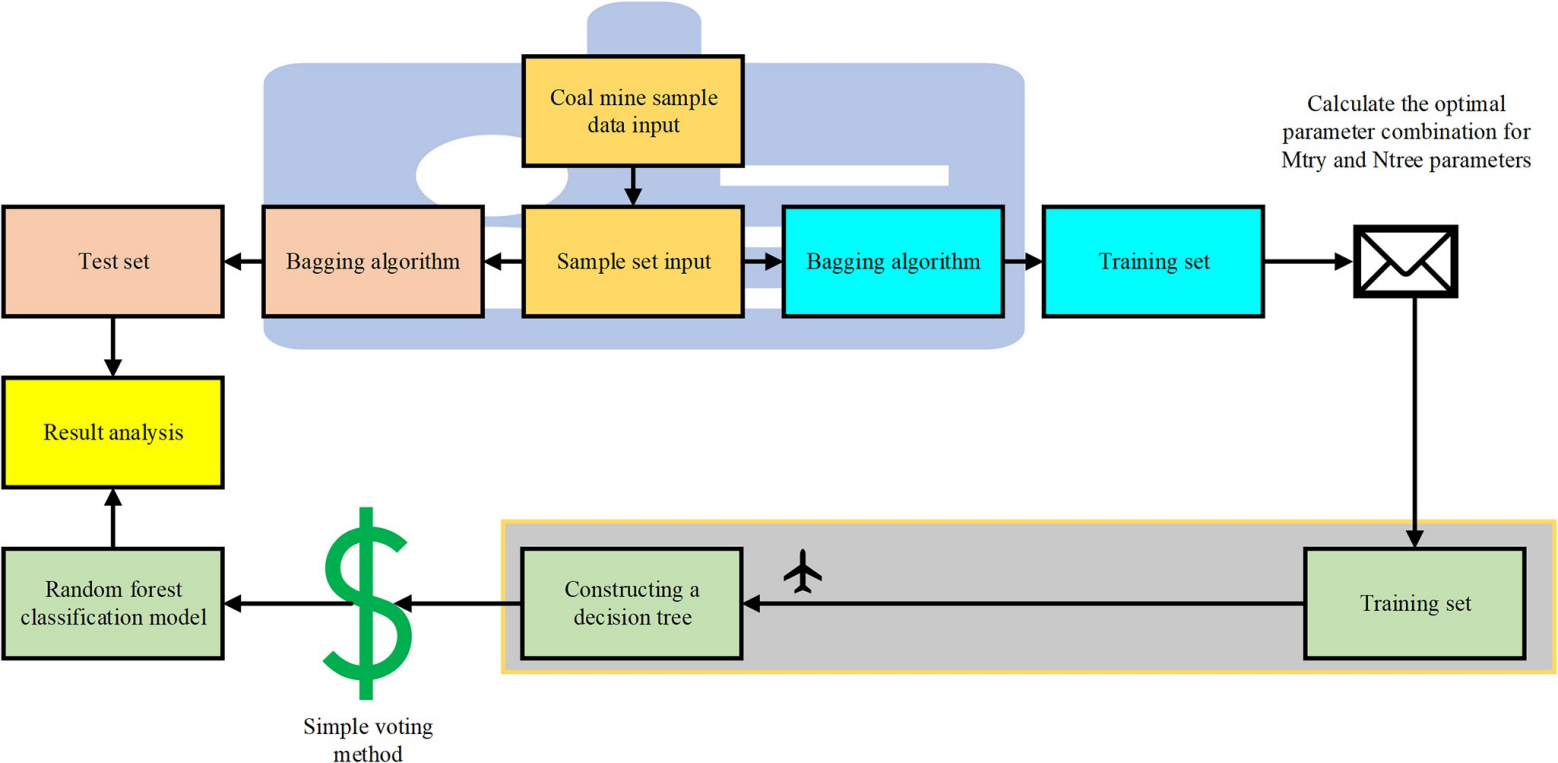
Optimized process of the random forest algorithm.

The Mtry parameter determines the number of features that can be selected each time a decision tree node split. It is an important parameter that ensures the randomness of the random forest algorithm. A smaller Mtry value may cause the model to be easily over-fitted, because each tree uses fewer features. The larger Mtry value increases the diversity of the model and reduces the risk of over-fitting, but it may also damage the accuracy of the model on the training set. This is also the difference between the decision trees in random forests and traditional decision tree algorithms. In traditional decision trees, the optimal feature is selected from the entire feature set as the splitting feature. However, in the decision tree splitting process of random forests, increasing the model parameter Mtry will reduce the base classifiers’ independence, thereby decreasing the accuracy of the random forest classification model [36]. In this study, the ability to prevent over-fitting and the accuracy of the model on the training set are comprehensively considered, and the model performance under different Mtry values is evaluated by cross-validation to determine the best Mtry value. The parameter Ntree represents the number of decision trees constructed during the process of using the Bagging algorithm in the random forest as an ensemble learning algorithm. It indicates the number of decision trees built through the process of random sampling with replacement. Based on the theory of random algorithms, the generalization error of the classification model will gradually converge to a limit value as the number of decision trees increases. Excessively increasing the value of the model parameter Ntree will significantly increase the complexity of the random forest algorithm, resulting in a decrease in computational efficiency. Moreover, once the parameter Ntree exceeds a certain value, the complexity of the random forest will increase. In this study, the model performance and computing resources are comprehensively considered, the model performance under different Ntree values is evaluated by cross-validation, and the best Ntree value is determined. Therefore, it is necessary to determine the ideal value for Ntree from a specific parameter range to maximize the accuracy of the model classification. The optimized algorithmic process is shown in Fig 4:

## Experimental design

Intelligent mining system not only collects rich real-time data, but also has the characteristics of automation and intelligence, which can analyze and process the data in real time. Real-time data acquisition of intelligent mining system is a key part of mine safety monitoring and management. These real-time data come from many sources, including mine safety monitoring, personnel positioning and video monitoring system. In mine safety monitoring, sensor networks are widely used to monitor various key safety parameters, such as gas concentration, temperature, humidity and oxygen concentration. These sensors have various types, including gas sensors, smoke sensors and temperature sensors. They transmit data to the central data acquisition system through wireless network to ensure the accuracy and availability of data. The collected data usually need to be processed and cleaned, including data filtering, anomaly detection, and calibration. Personnel positioning system uses global positioning system or wireless positioning technology, such as wireless sensor network or radio frequency identification, to realize real-time positioning of miners. These technologies can be applied in outdoor, indoor and underground environments, and different positioning technologies can be selected according to requirements. Personnel positioning data is transmitted to the central system through wireless communication, and data processing and cleaning are usually needed to improve the positioning accuracy. In addition, the video monitoring system captures real-time images and videos in the mine through cameras and video recording equipment. These video data are transmitted to the central monitoring station through wireless network, and processed by encoding, decoding, compression and storage. In a word, the intelligent mining system collects multi-source real-time data through various sensors and technologies, including mine safety monitoring, personnel positioning and video monitoring. The integration and analysis of these data provide an important information base for gas explosion early warning and mine safety management.

In order to verify the random forest gas explosion early warning model constructed here, the dataset used in this study is the "Gas sensor array under dynamic gas mixtures" dataset. This dataset is used for researching gas sensor arrays and can be utilized for developing and testing gas explosion warning methods. The dataset was provided by researchers from the National Council for Scientific and Technical Research of Argentina. Each sample in the dataset contains 17 features, with 16 features corresponding to the resistance values of each metal oxide semiconductor gas sensor and 1 feature representing the reading of a temperature sensor. Data source: https://archive.ics.uci.edu/dataset/322/gas+sensor+array+under+dynamic+gas+mixtures, and it consists of 13,910 samples. The experimental environment of this study was set up with a CPU size of 2.5G, a server system version of Windows 7, a disk size of 12G, and an Apache-tomecat6 network. The following is a portion of the code used to run the model in the experiment:

#Training random forest classifier using optimal super parameters
RF_ Classifier = RandomForestClassifier (n_estimators = best_params [’n_estimators’],
Max_ Depth = best_ Params [’max_depth ’],
Min_ Samples_ Split = best_ Params [’min_samples_split ’],

The experiment sets the parameters uniformly, with a total of 100 decision trees, a minimum sample size of 30, a random seed number of 20, an auto feature selection, and a maximum depth of 16.

## Analysis of the gas explosion risk assessment model based on intelligent mining system and multivariate data analysis

### Parameter optimization results based on random forest gas explosion early warning model

**Fig 5:**
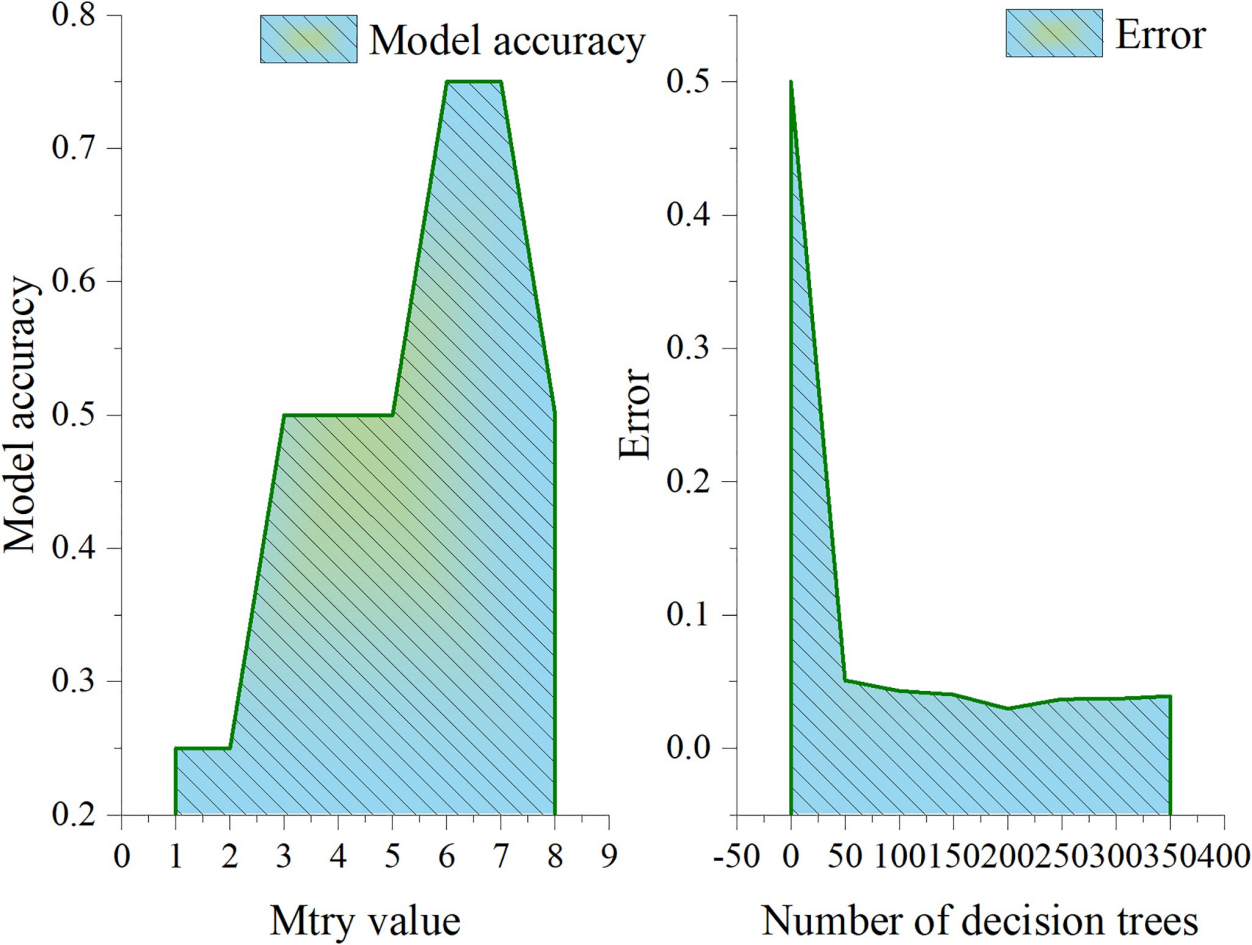
Parameter optimization results of random forest gas explosion early warning model.

This section analyzes the parameter optimization results of random forest gas explosion early warning model, as shown in Fig 5. Within the given range of Mtry and Ntree values, the accuracy and error of the model under different parameter combinations can be observed. Finally, the best Mtry and Ntree parameters can be obtained to optimize the model performance. The value range of Mtry parameter is 1 to 8, and the value range of Ntree parameter is 0 to 350, increasing every 50. The selection of the best Mtry and Ntree parameters is based on the comprehensive consideration of the accuracy and error of the model. Through Fig 5, when Mtry is 6, the accuracy of the model is the highest, which is 0.75. When Ntree is 200, the error of the model is 0.03. The best Mtry and Ntree parameters are 6 and 200 respectively, which is the best combination to optimize the performance of random forest gas explosion early warning model. Under this parameter combination, the model has the highest accuracy and the lowest error, which enables the model to better carry out gas explosion early warning and improve the prediction performance and reliability.

### Analysis of the evaluation results of the gas explosion risk assessment model

**Fig 6:**
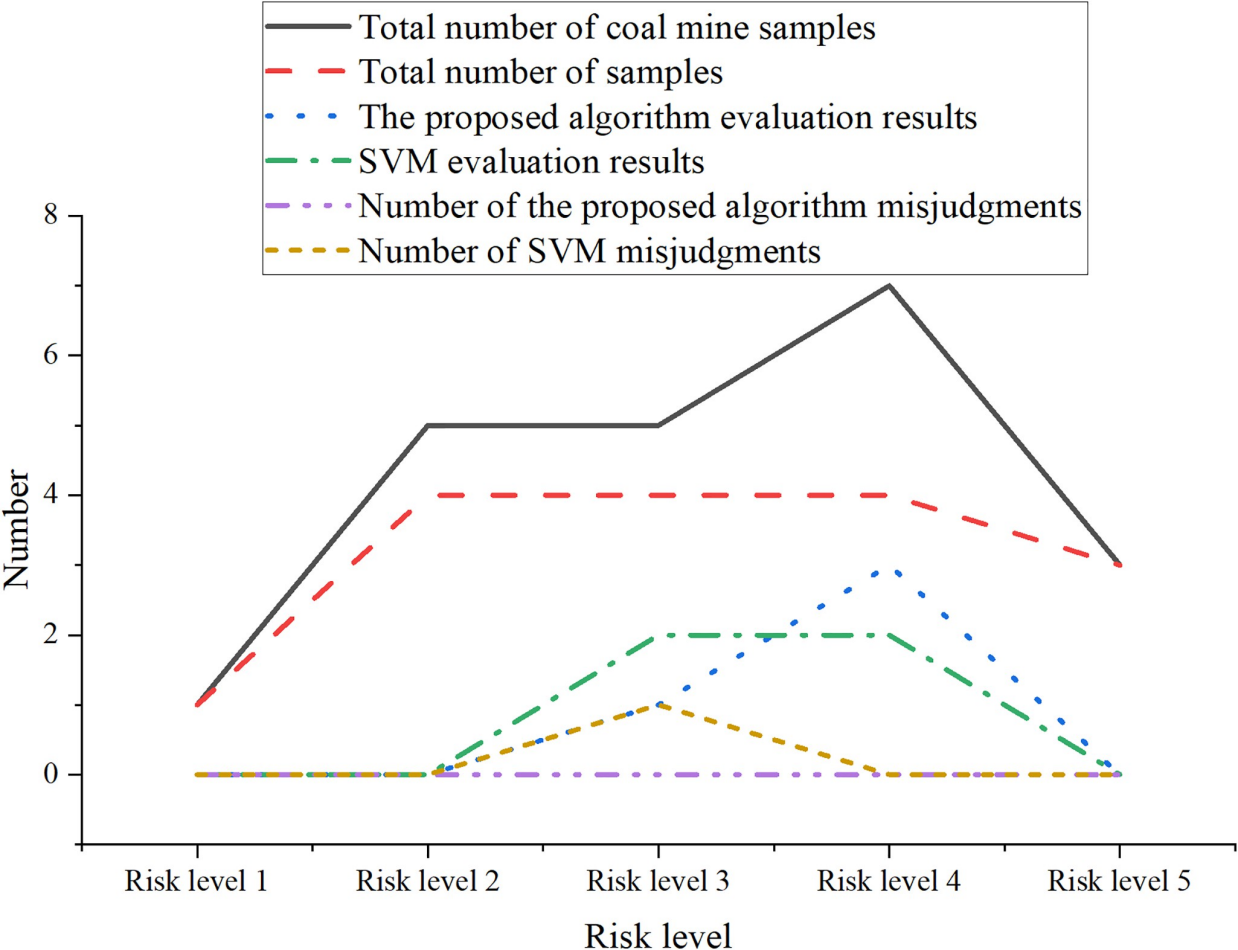
Comparative analysis of results between the optimized model and SVM.

Twenty samples were inputted into the optimized model proposed in this study and the SVM model. These samples were randomly divided, with 80% used as training samples and the remaining 20% used as test samples. The constructed optimized classification model and the SVM model were used to predict the classification of coal mine samples in the test set. This was done to evaluate the gas explosion risk of the samples in the test set. The comparison of the evaluation results between the optimized classification model and the SVM model is shown in Fig 6.

In Fig 6, the classification results of random forest model show high accuracy without any misjudgment. For different risk levels, its performance is very stable. In contrast, the performance of SVM model on the test set is slightly different. SVM model misjudged the coal mine samples with risk level 4, and wrongly classified a sample with risk level 4 as risk level 3. This leads to the number of misjudgments of SVM model is 1. To sum up, the random forest model shows higher classification accuracy on this set of test data, and there is no misjudgment, while the SVM model has misjudged in some cases. This emphasizes the performance advantage of random forest model in this particular problem. In order to evaluate the ability of the random forest classification model in gas explosion risk assessment based on a dataset of 87 coal mine samples, the performance of the optimized classification model and the SVM model were compared and analyzed. The specific comparison results are shown in Table 4.

**Table 4:** Comparison of accuracy between the optimized model and SVM.

Model	Model error	Test set risk level	Misjudgment number	Result accuracy
**The proposed algorithm**	0.1309	[4434]	0	100%
**LSTM**	0.1850	[4435]	1	92%
**RBF**	0.2023	[4432]	2	88%
**SVM**	0.2155	[4433]	1	75%

In Table 4, the model error of the algorithm (optimized model) proposed in this paper is 0.1309, the risk level of the test set is [4434], the number of misjudgments is 0, and the accuracy is 100%. This means that it completely correctly classifies all samples on the test set. The model error of SVM model is 0.2155, the risk level of test set is [4433], the number of misjudgments is 1, and the accuracy is 75%. This means that it has some classification errors on the test set. The misclassification rate of the optimized model is 0.1309, while that of the SVM model is 0.2155. This shows that the optimized model performs better in model error, and the relative error is lower than that of SVM model, with a difference of 0.0846. Based on the comprehensive analysis of the above results, it can be concluded that the model error of random forest algorithm in coal mine gas explosion evaluation is obviously better than SVM algorithm. The optimized model shows higher accuracy and reliability on the test set, which is of great significance for coal mine safety assessment.

### Comparative analysis of gas explosion disaster sub-system evaluation results

**Fig 7:**
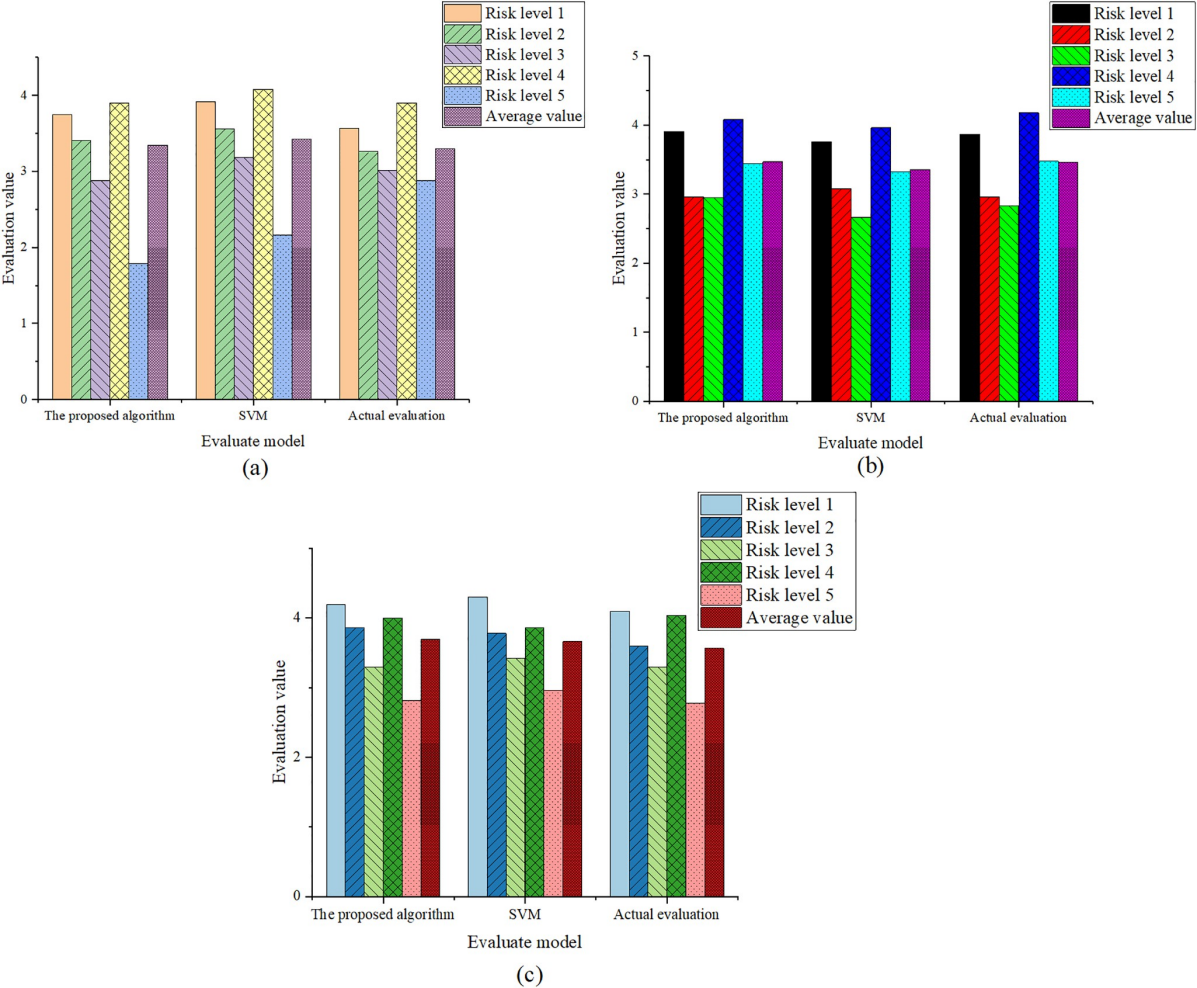
Comparative Results of Model Predictions (a) Comparison of Hazard Level Prediction in the Disaster-Causing Factor; (b) Comparison of Stability Prediction in the Disaster-Prone Environment; (c) Comparison of Fragility Prediction in the Disaster-Affected Body.

Treating coal mine gas explosion as a disaster system, the prediction and evaluation of the three secondary indicators are crucial. By utilizing the established random forest classification model, the prediction and evaluation of the secondary indicators in the test set are achieved. The predicted results are compared with the SVM model results and the actual evaluation, as shown in Fig 7.

In Fig 7, during the evaluation of the three sub-systems in gas explosion disasters, the optimized classification model in this study maintains a low level of model error in predicting the hazard level of the secondary indicator, the stability of the disaster-prone environment, and the fragility of the disaster-affected body. In the prediction of the hazard level of the disaster-causing factor, the evaluation values of the optimized classification model in this study are 3.75, 3.41, 2.88, 3.9, and 1.79, which are all lower than the evaluation values of 3.92, 3.56, 3.18, 4.08, and 2.16 obtained by SVM. Similarly, in the prediction of the stability of the disaster-prone environment, the evaluation values of the optimized classification model in this study are 3.91, 2.96, 2.95, and 4.08, which are also lower than the evaluation values obtained by SVM. The same trend is observed in the prediction of the fragility of the disaster-affected body, indicating the excellent performance of the optimized classification model in this study. In order to validate the rationality of the optimized model in this study, the misclassification rates between the model evaluation and the actual evaluation are calculated when the risk level is 5, as shown in Table 5.

**Table 5:** Comparison of misclassification rates between model evaluation and actual evaluation.

Model	Hazard prediction of disaster-causing factors	Prediction of the stability of pregnancy disaster environment	Vulnerability prediction of disaster-bearing bodies
**The proposed algorithm**	1.09	0.04	0.04
**LSTM**	0.92	0.03	0.05
**RBF**	0.88	0.06	0.07
**SVM**	0.72	0.16	0.18

Table 5 shows that the misclassification rates of the optimized classification model are 1.09 (for hazard assessment of disaster factors), 0.04 (for stability assessment of disaster environment) and 0.04 (for vulnerability of disaster-bearing bodies), while the relative errors of SVM model in the evaluation of three subsystems are 0.72, 0.16 and 0.18, respectively. Therefore, using the same coal mine sample set, the prediction results provided by the optimized random forest classification model are more in line with the actual situation compared with the results of SVM model. These results show that the prediction model based on optimized random forest has high applicability in the field of coal mine gas explosion early warning, especially in evaluating the danger of disaster factors, the stability of disaster environment, and the vulnerability of disaster carriers. This further emphasizes the importance of this research method to improve the accuracy and reliability of coal mine gas explosion early warning.

## Discussion

The experiment shows that when 87 samples are input into the model with unified parameter settings, the accuracy of the optimized classification model in this study reaches 100%, which is higher than the classification results of the SVM model. The misclassification rate of the optimized model in this study is 0.1309, while the SVM model’s relative error of sample classification is controlled above 2%. The model error of the optimized model in this study is 0.0846 lower than that of the SVM model, indicating that the optimized algorithm in this study performs better in terms of model error in gas explosion evaluation compared to the SVM algorithm. This is also attributed to the optimization of the two parameters, Mtry and Ntree, which improves the performance of the model. Meng et al. (2021) established a coal mining face safety early warning model based on fuzzy C-means clustering and back propagation neural network, which could realize the prediction and early warning of the working face safety. The neural network model optimized by genetic algorithm had better performance than the traditional back propagation artificial neural network model, and had higher prediction accuracy and convergence speed [4]. Different from their research, this study adopts random forest algorithm for early warning of gas explosion, and improves the model performance by optimizing parameters Mtry and Ntree. The results show that the optimization model in this study achieves 100% accuracy on the test set, which is much higher than the 75% accuracy of SVM model, indicating that the performance of random forest is excellent on this issue. Zhao et al. (2021) designed a coal mine underground hazard identification and early warning system based on random forest algorithm, and the average identification error of the system was only 4.1% [7]. This showed that the random forest algorithm had potential application in the field of coal mine safety. The research of this study also adopts random forest algorithm, but focuses on early warning of gas explosion. Although the problem areas are different, the results of this study also prove the effectiveness of random forest in the field of coal mine safety, especially in improving the accuracy of early warning of gas explosion. Li et al. (2022) thought that technical environment, working environment, organization management and hazard identification played a key role in predicting results [37]. The consideration of these factors was very important in coal mine safety assessment. This study focuses on the performance of the model, especially in the early warning of gas explosion, but these factors can also be combined in practical application to further improve the accuracy and practicability of the model. Li et al. (2023) put forward a model for dividing the roof richness of phosphate rock in karst area based on random forest. The research showed that when the number of decision trees was set to 200, the best performance was achieved, which was characterized by the highest accuracy of training samples and the smallest out-of-bag error [38]. This further confirms the effectiveness of random forest in different application fields. Related to this study, random forest is also used in the process of model optimization, and after the optimization of parameters Mtry and Ntree, higher accuracy and lower model error are finally achieved. In the comparative experiment of the evaluation results of gas explosion disaster sub-systems, the performance of the optimized classification model in this study is compared with that of the SVM model by evaluating the three sub-systems of gas explosion disaster. The model error of the optimized classification model in this study remains at a low level in evaluating the disaster-causing factor’s hazard level, the disaster-prone environment’s stability, and the disaster-affected body’s fragility. Specifically, in the prediction of the hazard level of the disaster-causing factor, the evaluation values of the optimized classification model in this study are significantly lower than the evaluation values of the SVM model. Similarly, in the prediction of the stability of the disaster-prone environment and the fragility of the disaster-affected body, the optimized classification model in this study also exhibits lower evaluation values. This result indicates that the optimized classification model in this study performs in gas explosion disaster early warning. Comparative experimental results demonstrate that the classification model based on the optimized random forest performs exceptionally well in gas explosion early warning. These results support the innovation and effectiveness of the coal mine explosion early warning method proposed in this study, based on intelligent mining systems and multi-dimensional data analysis.

## Conclusion

In order to improve the early warning capability of coal mine explosions, this study optimized the coal mine warning model through intelligent mining systems and multi-dimensional data analysis. On the one hand, the study established a gas explosion disaster early warning index system through data collection and analysis in the intelligent mining system. A coal mine gas explosion disaster early warning model was constructed based on the random forest classification algorithm. On the other hand, by optimizing the classification evaluation model through the setting of parameters Mtry and Ntree, the feasibility of the optimized model in this study was verified through experiments. The experimental results show that the optimized random forest model achieves 100% accuracy in predicting gas explosion disaster of coal mines, while the accuracy of SVM model is 75%. In addition, the model error of the optimization model in this study is 0.0846 lower than that of the SVM model. The optimized random forest model also maintains a low level of model error in evaluating the risk degree of disaster factors, the stability of disaster-prone environment and the vulnerability of disaster-affected bodies. In a word, this study improves the accuracy and reliability of early warning of coal mine gas explosion by combining intelligent mining system with advanced data analysis methods, which is expected to have a positive impact in the field of mine safety. This method can not only be applied to coal mine industry, but also provide valuable experience for safety early warning and risk assessment in other fields. This study does have some limitations, one of which is the limitation of the number of samples. This study is only based on 87 coal mine samples. This may affect the generalization ability and adaptability of the model to various situations. People should realize that coal mine safety management involves complex multi-dimensional factors, so future research should consider adding more samples to evaluate the model performance more comprehensively. Moreover, this study mainly focuses on the early warning of gas explosion, and does not cover other potential coal mine safety risks, such as mine fires and geological disasters. These factors are equally important in actual coal mine safety management. Future research can consider expanding the scope of research and in-depth study of early warning and management methods of different disaster types to improve the overall level of coal mine safety. In addition, people can further explore how to integrate intelligent mining system with modern technologies, such as artificial intelligence and big data analysis to build a more comprehensive and intelligent coal mine safety management system and improve the accuracy and efficiency of early warning. This will bring more possibilities and development space for research and practice in the field of coal mine safety.

## Supporting information

S1 Data (ZIP)
