# Supplementary material for: Gas explosion early warning method in coal mines by intelligent mining system and multivariate data analysis
Source: PLoS One. 2023 Nov 2;18(11):e0293814. doi: 10.1371/journal.pone.0293814 (PMC10621839; doi:10.1371/journal.pone.0293814)
Supplement: S1 Data — (ZIP) [file pone.0293814.s001.zip › data/code description.docx]

Code running environment: Windows 10

Running software: JetBrains PyCharm 2018.3.7 ×64

Running language software configuration: Python 3.8

Run the corresponding module to be loaded, Windows environment loading command:

pip install numpy

pip install pandas

pip install scikit-learn

Code description:

Python code is used to show how to optimize the early warning model of coal mine gas explosion by adjusting the parameters of random forest algorithm.

1. Firstly, the required libraries are imported, including random forest classifier, dataset splitting, accuracy evaluation and other commonly used machine learning libraries.

2. The parameter ranges of Mtry and Ntree are defined, where the range of Mtry is 1 to 8 and the range of Ntree is 0 to 350.

3. Two nested loops are used to traverse different combinations of Mtry and Ntree.

4. A random forest classifier is constructed in the loop, and the training data is used to fit the model.

5. The dataset is divided into training set and test set, with the ratio of 0.2 and random seed of 0.

6. The test set is predicted and the accuracy of the model is calculated.

7. In each iteration, the best parameters and the best accuracy are updated to find the best combination of Mtry and Ntree parameters.

8. Finally, the best parameters and the best accuracy are output.
